# A Preliminary Study on Hepatoprotective, Hypolipidemic and Aortic Morphometric Effects of Omega-3-Rich Fish Oil in Hypercholesterolemic Mice

**DOI:** 10.3390/ph17010072

**Published:** 2024-01-06

**Authors:** Ana Lina C. C. Sales, Maísa G. S. Primo, Renato S. Mello Neto, Ana Victória S. Mendes, Mariely M. Furtado, Joana Érica L. Rocha, José Otávio C. S. Almeida, José Vinícius S. França, Salmon R. Alencar, Ana Karolinne S. Brito, Luana O. Lopes, Márcia S. Rizzo, Ana Karina M. F. Lustosa, Paulo Humberto M. Nunes, Massimo Lucarini, Alessandra Durazzo, Daniel Dias Rufino Arcanjo, Maria do Carmo C. Martins

**Affiliations:** 1 Department of Biophysics and Physiology, Federal University of Piaui, Campus Ministro Petrônio Portella, Ininga, Teresina 64049-550, PI, Brazil; ana.lina123@gmail.com (A.L.C.C.S.); maisaguimaraessp@gmail.com (M.G.S.P.); renato.sampaio.mn@gmail.com (R.S.M.N.); victoriams18@hotmail.com (A.V.S.M.); marielymf@live.com (M.M.F.); ericarocha6@hotmail.com (J.É.L.R.); otavios.almeida@hotmail.com (J.O.C.S.A.); vinicius.sfranca@ufpi.edu.br (J.V.S.F.); salmonalencar@hotmail.com (S.R.A.); anakarolinnesb@hotmail.com (A.K.S.B.); phmnunes@ufpi.edu.br (P.H.M.N.); daniel.arcanjo@ufpi.edu.br (D.D.R.A.); 2 University Hospital, Federal University of Piauí, Campus Ministro Petrônio Portella, Ininga, Teresina 64049-550, PI, Brazil; luanalopesss@hotmail.com; 3 Department of Morphology, Federal University of Piaui, Campus Ministro Petrônio Portella, Ininga, Teresina 64049-550, PI, Brazil; marciarizzo@ufpi.edu.br; 4 Galeno Farmácia de Manipulação, Virgínia Regina Fortes Castelo Branco e Cia. Ltda., Teresina 64001-260, PI, Brazil; ana_lustosa@uol.com.br; 5 CREA-Research Centre for Food and Nutrition, Via Ardeatina 546, 00178 Rome, Italy; massimo.lucarini@crea.gov.it

**Keywords:** DHA, dyslipidemia, EPA, omega-3, supplementation, non-alcoholic fatty liver disease (NAFLD)

## Abstract

This study aims to evaluate the hepatoprotective, hypolipidemic and aortic morphometric effects of fish oil rich in omega-3 in hypercholesterolemic BALB/c mice. This is an experimental model that included 16 male BALB/c mice (*Mus musculus*) divided into three groups (G1 (standard commercial chow and 0.9% saline solution), G2 (hypercholesterolemic diet and 0.9% saline solution) and G3 (hypercholesterolemic diet and fish oil)) for 8 weeks. There was no significant difference in the treatment with omega-3-rich fish oil in the lipid profile (*p* > 0.05). In the histological analysis, group G2 detected the presence of hepatitis and liver tissue necrosis, but this was not observed in group G3. As for the morphometry in the light area of the vessel, the G1 group had a higher score (2.62 ± 0.36 mm^2^) when compared to G2 (2.10 ± 0.16 mm^2^) and G3 (2.26 ± 0.25 mm^2^) (*p* < 0.05). The vessel wall thickness did not differ between the groups (*p* > 0.05). It is concluded that supplementation with fish oil rich in omega-3 carried out in this study may have a protective effect on liver tissue, but it has not yet improved the lipid and morphometric profile. Despite this research being preliminary, it is a relevant study with future prospects for improving the doses of EPA and DHA in order to better elucidate the benefits of fish oil in models of dyslipidemia.

## 1. Introduction

Diets high in fat and cholesterol can contribute to the excessive accumulation of triglycerides (TG) in the liver, which, hydrolyzed into saturated free fatty acids, can overload the liver’s ability to metabolize them [1]. In addition, there is an environment of oxidative stress and inflammation that, together with the toxicity of FAFAs, can lead to the evolution of steatosis, non-alcoholic fatty liver disease (NAFLD), hepatocyte death and fibrosis [2]. The consumption of fish oil (FO) rich in omega-3 favors a reduction in the plasma concentration and content of hepatic triglycerides by decreasing hepatic lipogenesis and increasing the activity of lipoprotein lipase (LPL), accelerating the catabolism of VLDL and chylomicrons [3]. Although the hepatoprotective and lipid-lowering effects of omega-3 rich fish oil supplementation are still controversial, many studies reinforce its protective role in these parameters. Rocha et al. [4] supplemented male rats with omega-3-rich fish oil for 8 weeks and was able to improve liver tissue damage by reducing the amount of lipid vacuoles and liver triglyceride content compared to the group that did not receive the treatment. This preliminary study is therefore justified with the aim of evaluating the hepatoprotective, hypolipidemic and aortic morphometric effects of fish oil rich in omega-3 in hypercholesterolemia BALB/c mice.

## 2. Results

### 2.1. Weight Gain and Food Intake of the Treatment Groups

Data regarding initial weight, final weight, and daily food intake are shown in Table 1. The group supplemented with FO had a significantly higher average body weight compared to the other groups. At the end of the study, the control group (G1) exhibited weight gain, while groups G2 and G3 exhibited weight loss. Food intake was significantly higher in groups G1 and G3 than in group G2.

### 2.2. Effect of Omega-3 Fatty Acid Supplementation on Blood Glucose, Lipid Profile, and Liver Cytolytic Enzymes

**Figure 1:**
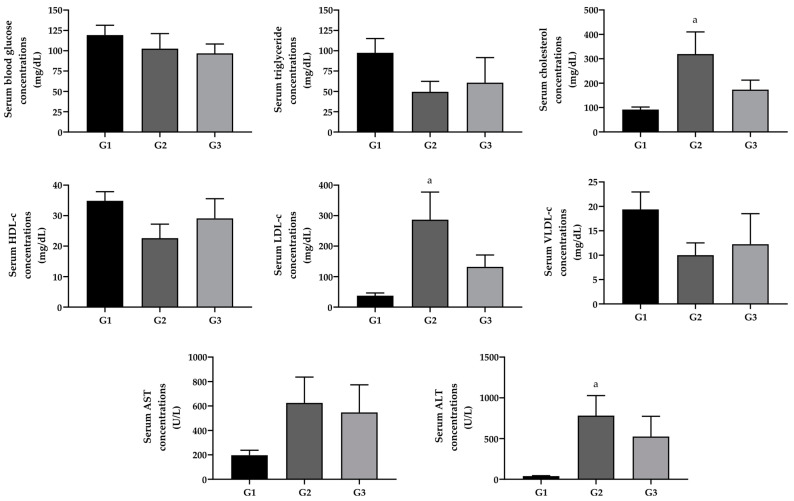
Serum concentrations of glycemia, TG, CT, HDL-c, LDL-c, VLDL-c AST, and ALT in mice in experimental groups G1 (standard diet + saline, *n* = 8), G2 (hypercholesterolemic diet + saline, *n* = 4) and G3 (hypercholesterolemic diet + FO, *n* = 4). Different letters represent a statistically significant difference (^a^ *p* < 0.05 when G1 is compared with G2). The data presented are expressed as mean and standard error of the mean using the Kruskal–Wallis test (*p* < 0.05) followed by the Dunn–Bonferroni post-test for TG, CT, HDL-c, LDL-c and VLDL-c and AST and one-way ANOVA followed by Tukey’s post-test (*p* < 0.05) for blood glucose and ALT.

As reported in Figure 1, there was no difference in fasting blood glucose between the studied groups. Regarding the serum lipid profile, TC and LDL-c exhibited significantly higher values in G2 than those reported in G1 (*p* < 0.05).

For liver cytolysis enzymes, ALT showed increased values for the G2 group when compared to the G1 group. Treatment with FO resulted in no change in lipid profile parameters and liver cytolysis enzymes (Figure 1).

### 2.3. Effect of Omega-3 Fatty Acid Supplementation on Oxidative Stress Biomarkers

Fish oil treatment did not result in a significant change in MPO activity when compared to the other groups in both plasma and liver. Regarding MDA, used as a marker of lipid peroxidation in liver homogenate, concentrations were lower in the G2 group when compared to the G3 group, indicating that FO supplementation promoted an increase in MDA concentrations.

**Figure 2:**
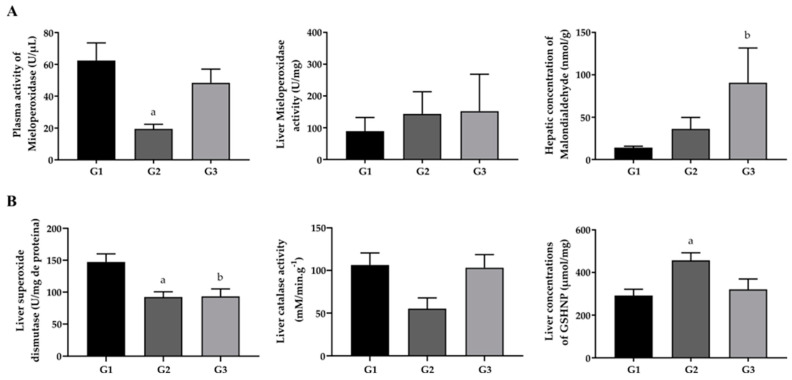
(**A**) Plasma and liver concentrations of MPO and MDA and mice in the experimental groups G1 (standard diet + saline, *n* = 8), G2 (hypercholesterolemic diet + saline, *n* = 4) and G3 (hypercholesterolemic diet + FO, *n* = 4). (**B**) Liver antioxidant enzyme activity SOD, CAT and hepatic concentration of GSHNP in mice in experimental groups G1 (standard diet + saline, *n* = 8), G2 (hypercholesterolemic diet + saline, *n* = 4) and G3 (hypercholesterolemic diet + FO, *n* = 4). Different letters represent a statistically significant difference (^a^ *p* < 0.05 when G1 is compared with G2; ^b^ *p* < 0.05 when G1 is compared with G3). The data presented are the mean and standard error of the mean with the application of the one-way ANOVA followed by the Tukey post-test (MPO; SOD, CAT and GSHNP) and the Kruskal–Wallis test followed by the Dunn–Bonferroni post-test (MDA) (*p* < 0.05). MPO = myeloperoxidase. MDA = malondialdehyde; SOD = superoxide dismutase; GSHNP = non-protein sulfhydryl groups; CAT = catalase.

GSHNP G2 showed significantly higher values compared to G3. On the other hand, the increase in GSHNP in the G2 group compared to the G1 group indicates that the hypercholesterolemic diet promoted an increase in the hepatic levels of GSHNP. SOD activity showed a reduction for animals G2 and G3 compared to G1 (*p* < 0.05) (Figure 2).

### 2.4. Effect of Omega-3 Fatty Acid Supplementation on the Lipid Content of Liver Tissue

**Figure 3:**
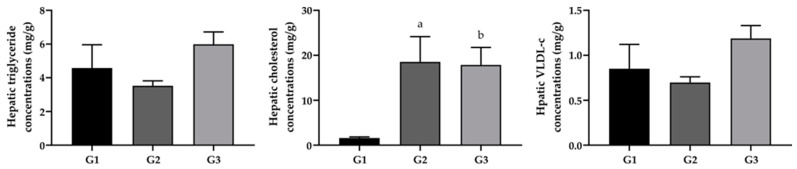
Lipid profile in the liver tissue of mice from experimental groups G1 (standard diet + saline, *n* = 8), G2 (hypercholesterolemic diet + saline, *n* = 4) and G3 (hypercholesterolemic diet + FO, *n* = 4). The data presented are the mean and standard error of the mean with the application of the Kruskal–Wallis test followed by the Dunn–Bonferroni post-test (*p* < 0.05). Different letters represent a statistically significant difference (^a^ *p* < 0.05 when G1 is compared with G2; ^b^ *p* < 0.05 when G1 is compared with G3).

The concentrations of TG, CT and VLDL-c extracted from the liver tissue of groups G1, G2 and G3 are represented in Figure 3. The group of animals that received a hypercholesterolemic diet and supplementation with FO (G3) showed a difference between CT in the liver tissue in relation to G1 and G2 (*p* < 0.05). No significant difference was observed between the groups in terms of the hepatic content of TG and VLDL-c.

### 2.5. Effect of Fish Oil Supplementation on the Morphological, Morphometric and Histological Aspects of the Liver

**Figure 4:**
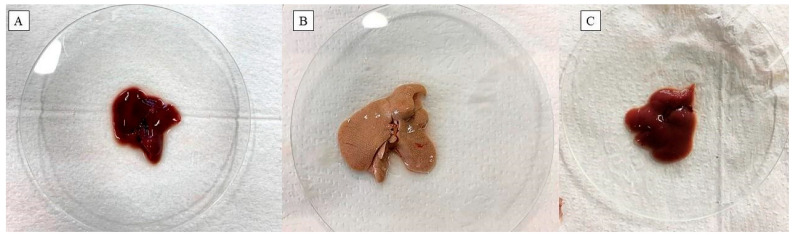
Macroscopic appearance of livers from mice submitted to a hypercholesterolemic diet. (**A**) standard diet + saline; (**B**) hypercholesterolemic diet + saline; (**C**) hypercholesterolemic diet + FO.

The liver of animals that received a hypercholesterolemic diet and 0.9% saline solution (G2) showed a pale color, indicating the accumulation of lipids. The alterations were focal and less evident for the animals that received a hypercholesterolemic diet and FO (G3). The liver of animals in G1 did not show macroscopically visible alterations (Figure 4).

**Figure 5:**
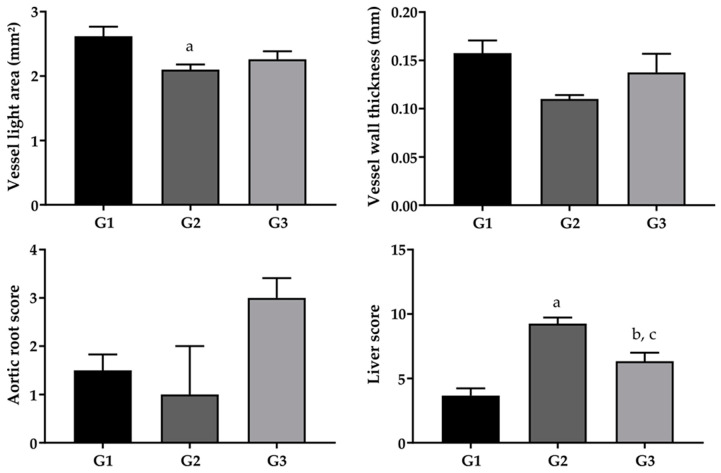
Vessel wall thickness, vessel light area, aortic root and liver score of experimental groups G1 (standard diet + saline, *n* = 8), G2 (hypercholesterolemic diet + saline, *n* = 4) and G3 (hypercholesterolemic diet + FO, *n* = 4). The data presented are the mean and standard deviation with the application of the one-way ANOVA followed by Tukey’s post-test (*p* < 0.05). Different letters represent statistically significant difference (^a^ *p* < 0.05 when G1 is compared with G2; ^b^ *p* < 0.05 when G1 is compared with G3; ^c^ *p* < 0.05 when G2 is compared with G3).

With regard to aortic root scores and vessel wall thickness, no statistically significant differences were found between the studied groups. However, for the vessel lumen area, the G1 group had a higher score when compared to G2 and there were no differences between the G1 and G3 and G2 and G3 groups (Figure 5).

The analysis of the steatosis scores of the hepatic tissues of both groups on the HC diet showed high levels of inflammation and steatosis (Figure 5 and Table 2), with a significant difference between these groups and the negative control (G1).

**Figure 6:**
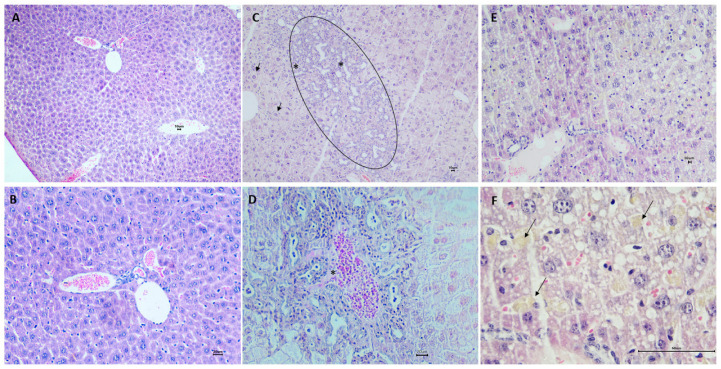
Photomicrographs of mouse liver tissue from the standard diet (**A**,**B**), hypercholesterolemic diet + saline (**C**,**D**), and hypercholesterolemic diet + FO (**E**,**F**). Hematoxylin–Eosin (H.E.) stain (scale bar: 10 µm (**A**,**C**,**E**), 20 µm (**B**,**D**), and 60 µm (**F**)). (**A**) Histological section of the normal liver with hepatocytes arranged in branching plates separated by capillary sinusoids. (**B**) Detail of (**A**) showing the portal triad. (**C**) The image shows an increased volume of hepatocytes (ballooning degeneration—arrows), mononuclear inflammatory cell infiltrates (*) adjacent to the area of bile ductule proliferation (circle). (**D**) Area showing hemorrhage and cholangitis with secondary inflammation (edema, neutrophils, mononuclear cells) around bile ductule proliferation (*). On the right, there is necrosis of hepatocytes and some hepatocytes with steatosis on the left. (**E**) In this section, hepatocytes are arranged in cords, and there is Kupffer cell hyperplasia. (**F**) Detail of E showing hepatocytes with microvacuolar degeneration and some with cytoplasmic accumulation of bile pigments (arrows). Analyzes were performed on all animals. Each image represents only one animal from each group.

Figure 6A,B show liver tissue from the standard diet group, in which hepatocytes show a normal format. In G2, as shown in Figure 6C,D, hepatocytes of varying sizes and volumes can be observed as a result of cytoplasmic microvacuolation with diffuse distribution throughout the tissue, which is suggestive of microvesicular steatosis (Table 2).

In G3, hepatocytes showed cytoplasmic microvacuoles diffusely distributed throughout the tissue, suggestive of microvesicular steatosis. In addition, there was moderate hyperplasia of Kupffer cells and the presence of intrahepatic cholestasis, with the presence of bile pigments (Figure 6E,F; Table 2).

## 3. Discussion

This study is one of the first to evaluate the hepatoprotective, lipidemic and aortic morphometric effect of FO supplementation rich in omega-3 in mice of the BALB/c species with this experimental model for the induction of hypercholesterolemia and a treatment time of 8 weeks. In addition, the parameters of lipid peroxidation and antioxidants have also been evaluated. There was an improvement in liver damage with a decrease in liver risk with no effect on the lipid profile, aortic morphometric parameters, lipid peroxidation and antioxidants.

FO treatment, although it resulted in weight loss, had no change in feed intake. Some studies have consistently reported greater thermogenesis after consuming unsaturated fats compared to saturated fats [5,6]. Thus, it is possible that the dietary composition of fatty acids may have influenced the metabolism of the animals, possibly affecting weight control. In this study, treatment with FO did not produce changes in the lipid profile. Likewise, Mendes Furtado et al. [7] did not observe a reduction in any parameter of the lipid profile in Wistar rats treated with omega-3 for 8 weeks when compared to the group treated with the HC diet.

Although supplementation with FO did not have a benefit on TG concentrations, it is worth mentioning the mechanisms of FO on TG. It is known that EPA and DHA can be incorporated into membrane phospholipids and participate in mechanisms associated with the regulation of lipid metabolism, mainly in the reduction in TG through the increase in hepatic β-oxidation, causing a reduction in the availability of fatty acids for its synthesis. In addition, they act in the regulation of apolipoprotein B-100, essential for the production of VLDL-c [8]. Lin et al. [9] reported that these effects can be suppressed with diets supplemented with 0.5% cholesterol.

The analysis of the lipid content in the hepatic tissue showed effects of omega-3 supplementation by administering FO on the TC content in relation to the group that consumed the HC diet. A similar result was found by Lima Rocha et al. [4] for the liver TC and TG content at the end of 8 weeks of FO supplementation when compared to the HC diet group.

There is a hypothesis that EPA and DHA have differential and divergent effects on metabolic pathways, which, in this study, would explain why FO did not have beneficial effects on the serum lipid profile and on the content of TG and VLDL-c in the liver tissue [10]. From this, it is believed that DHA reduces the synthesis of ApoC3, through hepatic transcription factors such as FOX-O1, resulting in greater hydrolysis of VLDL-c and, consequently, greater conversion of LDL into larger and less atherogenic particles [11,12].

Furthermore, they observed that DHA may be more effective than EPA at reducing blood triglyceride concentrations through differential regulation of lipogenic pathways, TG clearance, and inflammation, but these differences are not yet fully understood [13].

Regarding the serum concentrations of liver cytolysis enzymes, there was an increase in ALT in the HC diet group without supplementation when compared to the control group. This increase in liver enzymes in models of hypercholesterolemia indicates a probable onset of lesions such as non-alcoholic fatty liver disease and fibrosis [14]. In this study, the presence of hepatitis and necrosis in the liver tissue of mice in the HC diet group, but not in the group treated with FO, corroborates the changes in ALT.

Histologically and macroscopically, FO supplementation in this study appeared to ameliorate liver damage caused by an HC diet. This can be observed with an increase in Kupffer cells and by the less evident changes seen with the naked eye. Thus, FO supplementation seems to contribute to the regulation of hepatic lipogenesis through the inhibition of transcription factors associated with lipogenesis, increased expression of proteins linked to HDL transport and insulin modulation, which are increased with the consumption of a diet high in saturated fatty acids [15].

In this research, supplementation with FO did not show reduced values for lipid peroxidation markers such as MDA and MPO in the hepatic tissue, nor did it increase the hepatic concentrations of SOD, CAT and GSHNP when compared to those who did not receive treatment with FO. The explanation for this is based on the hypothesis of oxidation of polyunsaturated fatty acids and its relationship directly proportional to the degree of unsaturation. EPA and DHA are susceptible to oxidation due to multiple double and conjugated bonds and some causes that contribute to this are thermal oxidation, auto-oxidation and photo-oxidation [16]. The oxidation of EPA and DHA can trigger an increase in reactive oxygen species (ROS) with the presence of a greater amount of oxidized LDL, and this can influence the amount used in supplementation [17].

The probable oxidation of EPA and DHA in this study directly impacted the antioxidant system with decreased values; however, it is seen that the metabolic differences between EPA and DHA and their proportions can also interfere with the antioxidant potential. Nunzio and Bordini [18] verified the differences between polyunsaturated fatty acids in human hepatoma cells and observed that cells supplemented with DHA obtained a better antioxidant defense with SOD, GSH and GPx markers than those supplemented with EPA. However, it is still necessary to understand the effects of these nutrients in pathological conditions such as dyslipidemia [19].

Some limitations, such as the number of deaths in hypercholesterolemic groups, either treated with FO or not, as well as the duration of treatment may represent a limitation for marked general observations on the experimental model of dyslipidemia employed in this study. On the other hand, those issues do not invalidate this research because it is a study with preliminary data and future perspectives of improvements to these limiters. Nevertheless, its benefit is evident in the conditions of the present study, in relation to the improvement in the macroscopic and histological aspects of the liver, as well as in the reduction in the accumulation of lipids and inflammatory cells in this organ.

## 4. Materials and Methods

### 4.1. Experimental Model of Dyslipidemia

Sixteen male BALB/c mice (*Mus musculus*) were maintained at a controlled temperature of 24 ± 2 °C with a 12:12 h dark:light cycle and free access to food and water. The hypercholesterolemic diet was based on the methodology of Afonso et al. [20] with the addition of 0.5 g of cholesterol and 0.25 g of cholic acid (Inlab Confiança^®^, São Paulo, Brazil) for each 100 g of standard feed powder (Presence^®^, São Paulo, Brazil).

**Figure 7:**
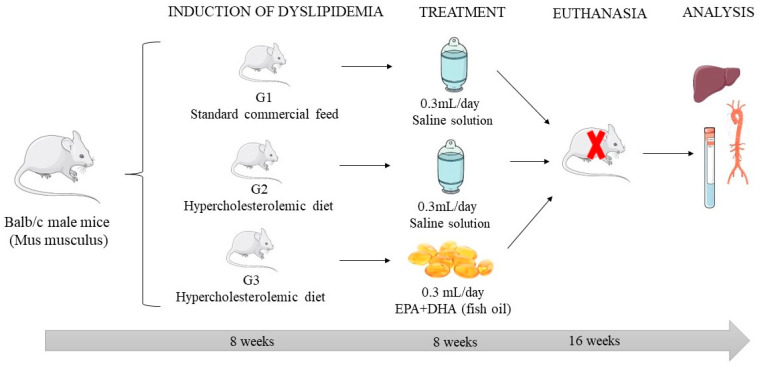
Experimental design of dyslipidemia induction and fish oil supplementation (EPA + DHA).

The animals were randomly distributed into three groups: G1 (standard commercial feed and 0.3 mL/day of 0.9% saline solution; *n* = 8), G2 (hypercholesterolemic diet and 0.3 mL/day of 0.0% saline solution; 9%; *n* = 4) and G3 (hypercholesterolemic diet and fish oil 0.3 mL/day; *n* = 4). The treatment was given once a day. The contents of FO (Infinity Parma^®^, São Paulo, Brazil) and saline solution were aspirated and administered orally (gavage). Dyslipidemia was induced by means of a hypercholesterolemic diet, offered ad libitum, for 8 weeks to the G2 and G3 groups. The G1 group was maintained with free access to standard commercial feed. After the induction period, the animals underwent treatments for 8 weeks (Figure 7). For this study, male animals were chosen in order to minimize physiological and hormonal interference that occurs with female animals; the use of female mice would require a larger number of animals due to the fluctuating hormonal concentrations in females since high variability can make data interpretation more difficult.

For humans, the recommendation for fish oil supplementation is approximately 1–3 g/day to obtain benefits against cardiovascular diseases [21,22,23]. Considering the straight body weight range of the animals used in our experimental protocols, in this research, we decided to use 0.3 mL of FO (EPA 57 mg/day; DHA 36 mg/day with a total of 93 mg of omega-3) per animal. This average daily consumption in rats, when subjected to a dose conversion for humans based on body surface area [24], is in line with the recommendations for beneficial supplementation against cardiovascular diseases as recommended by the American Heart Association [21], the Brazilian Society of Cardiology [22] and the European Society of Cardiology [23].

### 4.2. Determination of the Chemical Composition of the Diets

The evaluation of the proximate composition of the diets used (standard and hypercholesterolemic) was carried out by determining the moisture (926.12), ash (900.02), lipid (920.39), protein (991.20), and carbohydrate (obtained by difference/protocol number does not apply) contents using the methods recommended by the Association of Official Analytical Chemists (AOAC) [25].

It was found that the lipid values were significantly higher for the hypercholesterolemic diet used to induce dyslipidemia and that the energy and protein values were lower when compared to those obtained in the commercial standard diet (*p* < 0.05). There were no differences in carbohydrate content between the two diets (Table 3).

### 4.3. Assessment of Food Intake and Body Weight

The assessment of daily food intake was performed by weighing the remaining diet daily and subtracting it from the total amount of food available in the cage the previous day, thereby determining the total intake of the group of animals in the cage. The average amount of feed consumed per animal was obtained by dividing the total value obtained in each cage by the number of animals per box. Body weight values were obtained with the aid of a digital scale (Western Weighing Technologies Inc., Ferndale, WA, USA). Measurements were taken every 3 days during the entire experiment, and body weight gain was analyzed every 7 days [26].

### 4.4. Blood and Tissue Collection

At the end of the study, the animals were euthanized with intraperitoneal injection of 100 mg/kg sodium thiopental after 12 h of fasting. Blood was collected by cardiac puncture and distributed in tubes containing ethylenediaminetetraacetic acid (EDTA) or coagulation activator and processed according to the methods proposed by Whitehouse et al. [27].

### 4.5. Determination of Biochemical Parameters

The serum glucose, total cholesterol (TC) bound to high-density lipoprotein (HDL-C), and TG were determined using the enzymatic colorimetric method in an automated analyzer (LabMax Plenno; Labtest, Lagoa Santa, MG, Brazil). LDL-c was obtained using the formula of Friedewald, Lavy, and Fredrickson [28], where the very low-density lipoprotein (VLDL-c) consisted of an estimate of TG/5. All concentrations were expressed in mg/dL.

Concentrations of alanine aminotransferase (ALT) and aspartate aminotransferase (AST) were determined with a colorimetric method using an automated analyzer (LabMax Plenno; Labtest), and the results were expressed as U/L.

### 4.6. Determination of Oxidative Stress Biomarkers

#### 4.6.1. Determination of Myeloperoxidase (MPO) Activity

The measures of MPO activity in the plasma and liver were based on the oxidation rate of the substrate o-dianisidine in the presence of H_2_O_2_ and evidenced by the change in absorbance measured at 450 nm based on Bradley et al. [29]. The results were expressed as MPO per microliter of plasma sample (U/µL) or liver sample in (U/mg of liver).

#### 4.6.2. Determination of the Malondialdehyde (MDA) Concentration

MDA concentrations were determined by the production of substances reactive to thiobarbituric acid (TBARS) as described by Ohkawa et al. [30] with adaptations.

The results were read using a spectrophotometer at wavelengths of 510 nm, 532 nm, and 560 nm, and the corrected absorbance was calculated using the proposed formula to minimize the interference of the heme pigments and hemoglobin: ABS = 1.22 × [A_532_ − (0.56 × A_510_) + (0.44 × A_560_)] [31]. The results were expressed in nmol of MDA per g of liver tissue (nmol/g of liver).

### 4.7. Determination of the Antioxidant Activity in the Liver

#### 4.7.1. Superoxide Dismutase (SOD) Activity

The SOD activity in liver tissues was determined by the amount capable of inhibiting 50% of nitrite formation according to the method of Das, Samanta, and Chainy [32].

The results were read using a microplate reader at an absorbance of 543 nm and expressed as U/mg of protein. The protein concentration was determined using a colorimetric commercial kit according to the manufacturer’s instructions (Labtest^®^, Lagoa Santa, Brazil).

#### 4.7.2. Catalase Activity (CAT)

The determination of CAT activity was undertaken based on the method of Beutler et al. [33], who determined the CAT activity by quantifying the decomposition rate of hydrogen peroxide (H_2_O_2_) by the enzyme, which measures the decrease in optical density at 230 nm. The absorbance was read every 1 m for 6 min in a plate reader, and the results were expressed in mM/min·g^−1^.

#### 4.7.3. Determination of Non-Protein Sulfhydryl Group (GSHNP) Concentrations

The GSHNP measurements were undertaken in the liver according to the methodology of Habeeb et al. [34]. The results were expressed in µmol/mg of liver.

### 4.8. Extraction of Hepatic Lipids and Determination of Lipid Content

To obtain lipids from the liver tissue, we used the method of Folch et al. [35]. Finally, the lipids were dosed using an enzymatic colorimetric assay according to the manufacturer’s instructions [36,37]. The results were expressed in mg/g of liver.

### 4.9. Histopathological and Morphometric Analysis

The histopathological analysis of aorta, heart tissue root and liver tissue of male Balb/c mice (*Mus musculus*), which received fish oil supplementation, was based on qualitative (descriptive) and quantitative (measurements of vessel area and wall thickness) and semiquantitative (scores) data.

In the semiquantitative analysis (scores) in the liver tissue, alterations were evaluated for parameters of hemodynamic disorders, parenchyma disorders, Kupffer cell hyperplasia/hypertrophy and inflammatory cell infiltrate that received the following score classification (0): no alterations; (1): slight changes; (2): moderate changes; (3): severe alterations. The results obtained were expressed as cumulative scores from 0 to 12 [38,39].

### 4.10. Ethical Aspects

This study was approved by the Committee of Ethics in Animal Use of the Federal University of Piauí (CEUA/UFPI No. 446/18). The procedures carried out were in accordance with the ethical principles established for Animal Experimentation by the National Council for Animal Experimentation Control (CONCEA) [40].

### 4.11. Data Analysis

Data normality was verified using the Shapiro–Wilk test. To compare the two-group means, we used Student’s *t* test or the Mann–Whitney U test. One-way ANOVA followed by Tukey’s post-test was applied for parametric data, or the test Kruskal–Wallis followed by Dunn–Bonferroni post-test for non-parametric data, in order to compare the three-group means. For all tests, a confidence interval of 95% and *p* < 0.05 was adopted.

## 5. Conclusions

Supplementation with FO, at the dose and duration of treatment used, resulted in a hepatoprotective effect with a decrease in the risk of hepatic steatosis. There was no improvement in aortic lipid and morphometric parameters after intervention with FO. Despite the benefits of FO being reported in several studies, there are differences between the doses, composition and duration of interventions necessary to produce beneficial effects. Thus, further studies with different doses, higher or lower than those used in this preliminary study, could help to elucidate the potential properties of this supplementation.

## Figures and Tables

**Table 1:** Weight gain and food intake of experimental groups G1, G2 and G3.

Parameters	G1 Mean ± SEM	G2 Mean ± SEM	G3 Mean ± SEM
Starting weight (g) *	36.75 ± 0.95 ^a^	39.00 ± 1.27 ^a^	44.41 ± 1.84 ^b^
Final weight (g) *	41.62 ± 2.29 ^a^	29.50 ± 2.10 ^b^	27.33 ± 2.18 ^b^
Total weight gain (g) *	4.87 ± 2.08 ^a^	−6.75 ± 2.81 ^b^	−14.25 ± 2.01 ^c^
Daily food intake (g/day) *	5.24 ± 0.30 ^a^	4.13 ± 0.03 ^b^	4.87± 0.17 ^a^

SEM = standard error of the mean; (*n* = 4–8); G1 = standard ration + saline (16 s); G2 = hypercholesterolemia diet + saline (16 s); G3 = hypercholesterolemia diet + FO (15 s). Different letters represent a statistically significant difference (*p* < 0.05) between the groups for the variable presented in the line considered. * One-way ANOVA followed by Tukey’s post-test.

**Table 2:** Characterization of the proximate composition of the standard and hypercholesterolemia diet (HC) used in the study.

Groups	Hemodynamic Disorders	Parenchyma Disorders	Hyperplasia/Kupffer Cell Hypertrophy	Inflammatory Cell Infiltrates
G1	1	1	1	1
G2	2	3	2	3
G3	1	1	3	1

Caption: G1: standard diet + saline; G2: hypercholesterolemic diet + saline and G3: diet hypercholesterolemic + FO; (1): slight changes; (2): moderate changes; (3): severe changes to the parameters evaluated.

**Table 3:** Characterization of the proximate composition of the standard and hypercholesterolemiant diet (HC) used in the study.

Components	Standard Diet Mean ± SD	Diet HC Mean ± SD	*p* Value
Carbohydrates (g/100 g) ^1^	40.64 ± 0.83	43.63 ± 0.41	0.050
Protein (g/100 g) ^1^	39.53 ± 0.98	37.51 ± 0.42	0.046 *
Lipid (g/100 g) ^2^	2.12 ± 0.08	3.22 ± 0.09	0.000 *
Moisture (g/100 g) ^2^	8.47 ± 0.16	6.04 ± 0.13	0.000 *
Ashes (g/100 g) ^2^	9.24 ± 0.29	9.60 ± 0.21	0.156
VET (kcal/100 g) ^2^	339.79 ± 7.97	353.53 ± 4.13	0.000 *

SD = standard deviation; VET = total energy value; HC = hypercholesterolemiant; ^1^ Mann–Whitney U test; ^2^ Student’s *t* test for independent samples (* *p* < 0.05). Analysis performed in triplicate.

## Data Availability

Data will be available under request.
